# 3D Printed Robot Hand Structure Using Four-Bar Linkage Mechanism for Prosthetic Application

**DOI:** 10.3390/s20154174

**Published:** 2020-07-27

**Authors:** Mohamad Aizat Abdul Wahit, Siti Anom Ahmad, Mohammad Hamiruce Marhaban, Chikamune Wada, Lila Iznita Izhar

**Affiliations:** 1 Department of Electrical and Electronics Engineering, Faculty of Engineering, Universiti Putra Malaysia, Serdang, Seri Kembangan, Selangor 43400, Malaysia; mhm@upm.edu.my; 2 Malaysian Research Institute on Ageing, Universiti Putra Malaysia, Serdang, Seri Kembangan, Selangor 43400, Malaysia; 3 Department of Electrical & Electronic Engineering, Faculty of Engineering & Built Environment, Universiti Sains Islam Malaysia, Bandar Baru Nilai, Nilai, Negeri Sembilan 71800, Malaysia; 4 Graduate School of Life Science and Systems Engineering, Department of Human Intelligence Systems, Institute of Technology Kyushu, 2-4 Hibikino, Wakamatsu-ku, Kitakyushu-shi, Fukuoka 808-0196, Japan; wada@brain.kyutech.ac.jp; 5 Department of Electrical and Electronic Engineering, Universiti Teknologi PETRONAS, Seri Iskandar 32610, Malaysia; lila.izhar@utp.edu.my

**Keywords:** 3D printed, four-bar linkage mechanism, prosthetic hand, robot hand structure, static analysis, motion capture analysis

## Abstract

Trans-radial prosthesis is a wearable device that intends to help amputees under the elbow to replace the function of the missing anatomical segment that resembles an actual human hand. However, there are some challenging aspects faced mainly on the robot hand structural design itself. Improvements are needed as this is closely related to structure efficiency. This paper proposes a robot hand structure with improved features (four-bar linkage mechanism) to overcome the deficiency of using the cable-driven actuated mechanism that leads to less structure durability and inaccurate motion range. Our proposed robot hand structure also took into account the existing design problems such as bulky structure, unindividual actuated finger, incomplete fingers and a lack of finger joints compared to the actual finger in its design. This paper presents the improvements achieved by applying the proposed design such as the use of a four-bar linkage mechanism instead of using the cable-driven mechanism, the size of an average human hand, five-fingers with completed joints where each finger is moved by motor individually, joint protection using a mechanical stopper, detachable finger structure from the palm frame, a structure that has sufficient durability for everyday use and an easy to fabricate structure using 3D printing technology. The four-bar linkage mechanism is the use of the solid linkage that connects the actuator with the structure to allow the structure to move. The durability was investigated using static analysis simulation. The structural details and simulation results were validated through motion capture analysis and load test. The motion analyses towards the 3D printed robot structure show 70–98% similar motion range capability to the designed structure in the CAD software, and it can withstand up to 1.6 kg load in the simulation and the real test. The improved robot hand structure with optimum durability for prosthetic uses was successfully developed.

## 1. Introduction

Malaysia is a developing country with about 32.6 million population in 2019 [1], and the population is estimated to reach up to approximately 38 million in 2040. The World Health Organization (WHO) stated that about 0.5% of the population of a developing country have a disability that requires a prosthesis/orthosis and related rehabilitation services [2]. This will be more than 200 thousand people in 2040. This estimation indicates that research in prosthetic device development is of paramount importance to support the increasing demand for improved and affordable upper and lower extremity prostheses. In the literature, there are many studies on the development of a prosthesis device, especially the robotic hand that resembles the human hand and its function. The human hand is a complicated part in a human body to be duplicated due to its delicate and complex structure that enables a wide variety of functions, including gross and fine motor movements. In order to achieve the goal, the structural design has become the critical perspective that needs to be taken into account. It is because it majorly affects the robot hand movement capability; the durability and variation of the postures can be done by the robot hand for daily life use.

There are some sophisticated commercial robot hands available in the market today, such as Vincent Hand by Vincent Systems, iLimb by Touch Bionics, iLimb Pulse by Touch Bionics, Bebionic hand range by RSL Steeper, and Michelangelo Hand by Otto Bock. However, the structures of these robot hands do not meet the human hand characteristics due to the lack of the finger joint used, where the distal interphalangeal joint is excluded from the finger structure [3].

Recently, the structural design of the robot hand using the wired-driven mechanism as the movement mechanism became popular among researchers [4,5,6,7,8,9]. However, it is found that the use of wire or flexible linkage in this approach gives inaccurate joint movement due to the mechanical properties of the cable. Its shape is effortless to change, and its length often extends every time it is used due to the elasticity of wires [10,11]. Moreover, some researchers have released a robot hand with better features, such as five fingers, that uses a four-bar linkage mechanism. Still, the fingers do not individually move, and the dimensions are larger than the average size of the human hand [12,13]. We also found a design that has similar characteristics as the one mentioned above, but the structure of the robot hand was built with a metal material [14]. However, the structure of the robot is weighty as compared to that of a plastic material and is thus not suitable to be used as the material for a prosthetic hand. Over the years, there has been advancement in terms of the use of new plastics and other materials such as carbon fiber in the construction of hand prosthetic structures and 3D printing technology is also in line with this demand [5,7,9,15]. Furthermore, detachable finger structure and mechanical stopper are crucial features, which are useful for finger replacement and joint protection purposes, respectively. Table 1 shows the existing robot hand devices available in both commercial and research fields, and their mechanical characteristics.

This paper outlines the improvements made to some features of the robot hand structure that have been improved from existing robot hand structural designs. Moreover, our proposed robot structure was built using 3D printing technology, as suggested by previous studies. Additionally, the structure of the robot being built was determined to ensure similarity in terms of mobility and durability, as specified in the structural design.

## 2. Materials and Methods

The development of the robot hand structure’s overall process consisted of three main stages: planning, design and simulation, and structure fabrication [27]. In the planning stage, the general characteristic information of structural design was collected, and this consisted of two phases: design behaviour and design specification. The design behaviour phase was the primary consideration in the development of the structural design of the robot hand, focusing on the actual hand behaviour such as the number of joints and movement capabilities, etc. In the design specification phase, structure limitation was investigated and improved features were identified and applied to the structural design.

The design and simulation stage was the detailed design stage whereby the most suitable structural elements in terms of the proportions, dimensions, and other specifications and estimates based on actual adult human hands were determined. Furthermore, the finalized structural design was visualised and constructed in a three-dimensional drawing using CAD software. Then, the durability of the finger structural design was simulated to calculate the safety factor of the structure through the static analysis technique.

The robot hand structure was constructed using 3D printing technology (UPBOX+ 3D Printer) with Acrylonitrile Butadiene Styrene (ABS) thermoplastic material in the fabrication. The fabricated hand structure’s movement capability and durability were then measured in a motion capture analysis and load test, respectively. The evaluations were done towards the fabricated robot structure to validate the durability obtained in the simulation and the movement capability similarity towards the designated range of motion (ROM) was obtained in the design stage.

### 2.1. Planning

#### 2.1.1. Design Behavior

**Figure 1:**
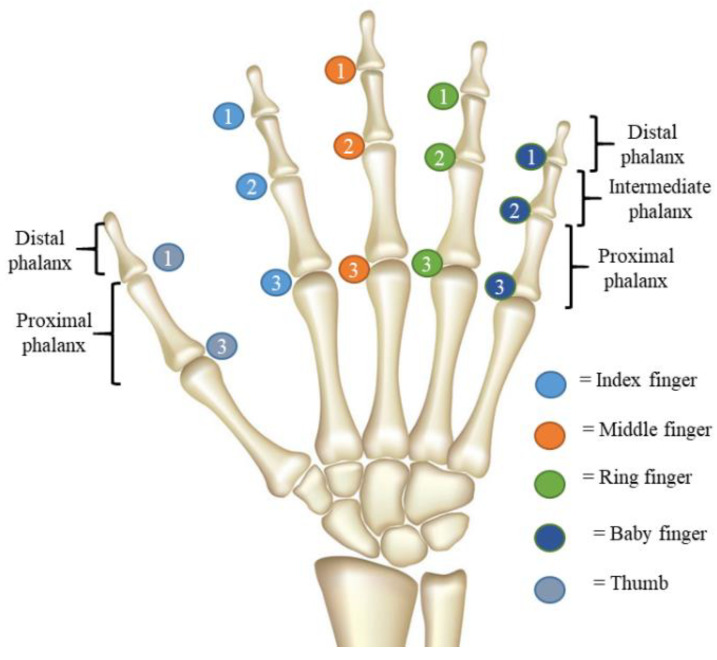
The anatomy of the human right-hand: 1 = Distal Interphalangeal, 2 = Proximal Interphalangeal, and 3 = Metacarpophalangeal.

**Figure 2:**
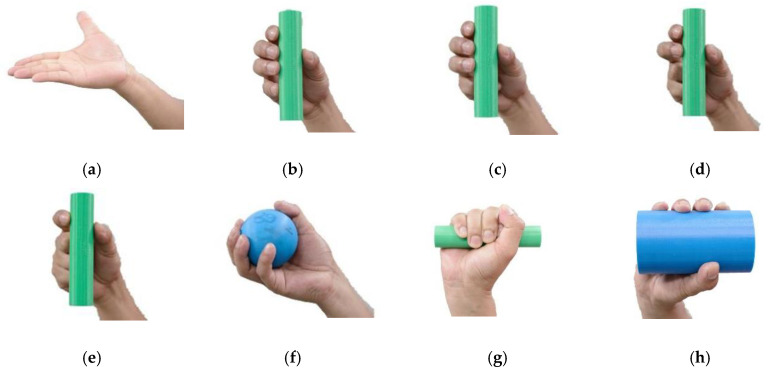
The hand postures for (**a**) opened hand palm, grasping: (**b**) Thumb–4 fingers, (**c**) Thumb–3 fingers, (**d**) Thumb–2 fingers, (**e**) Thumb–1 finger, and Prismatic prehensile: (**f**) Sphere object and (**g**) Prismatic prehensile, (**h**) Large cylinder object.

The actual human hand consists of five fingers: the thumb, index, middle, ring and baby fingers. Each finger has three bones: Distal Phalanx (DP), Intermediate Phalanx (IP) and Proximal Phalanx (PP), except the thumb has the DP and PP only. The connections between the bones are known as joints. They are the Distal Interphalangeal (DIP), Proximal Interphalangeal (PIP) and Metacarpophalangeal (MCP), respectively, as illustrated in Figure 1. The total number of joints is about 14 joints excluding the wrist joint, and these joints allow the hand to be bent when the muscle is contracted. The fingers are individually actuated by the muscles, which allow them to move as required. There are two muscle types known as the flexor and extensor. The flexor is the muscle used to reduce the angle between the bones on both sides of the joint, such as bending the fingers, and is also known as the flexion. The extensor muscle is used to increase the angle between the bone components and the motion, also known as extension. Either the contraction of the flexor or extensor will enable a variety of hand postures such the opened hand palm motion, Grasping: Thumb–4 fingers, Thumb–3 fingers, Thumb–2 fingers, Thumb–1 finger, and Prismatic prehensile: Sphere object, small object and large cylinder object and many more [28], as shown in Figure 2.

#### 2.1.2. Design Specification

**Figure 3:**
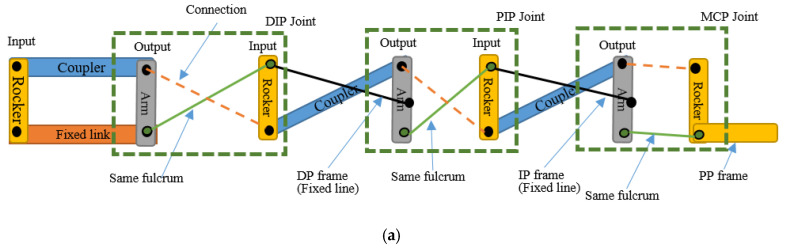

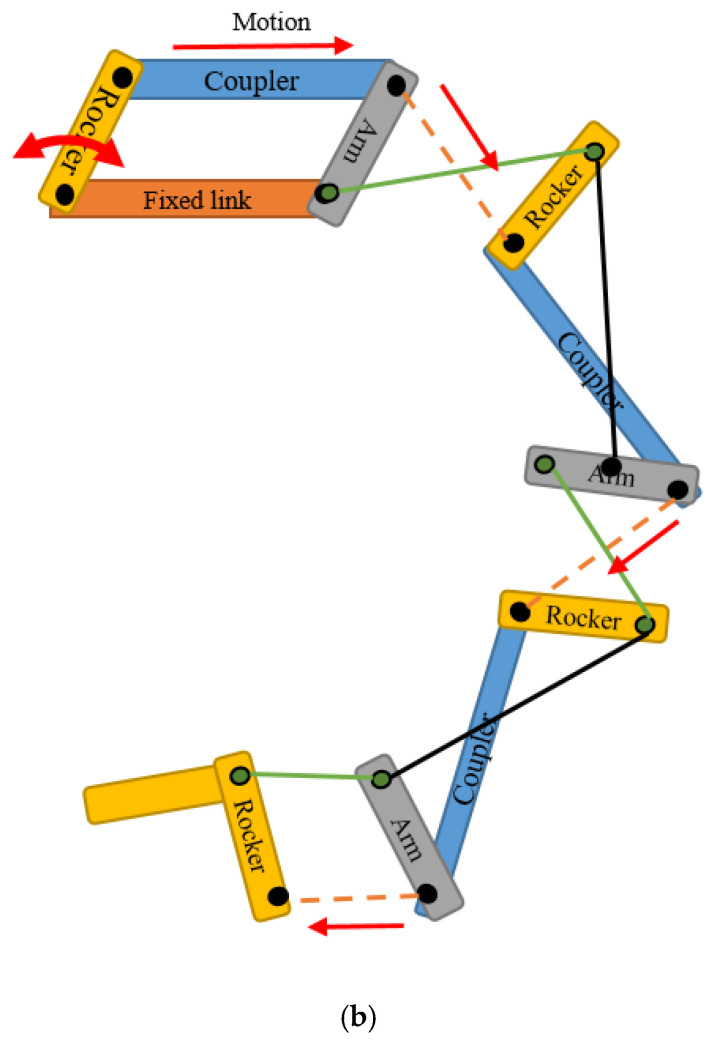
The finger robot structure: four-bar linkage mechanism diagram (**a**) extension motion, (**b**) flexion motion.

**Figure 4:**
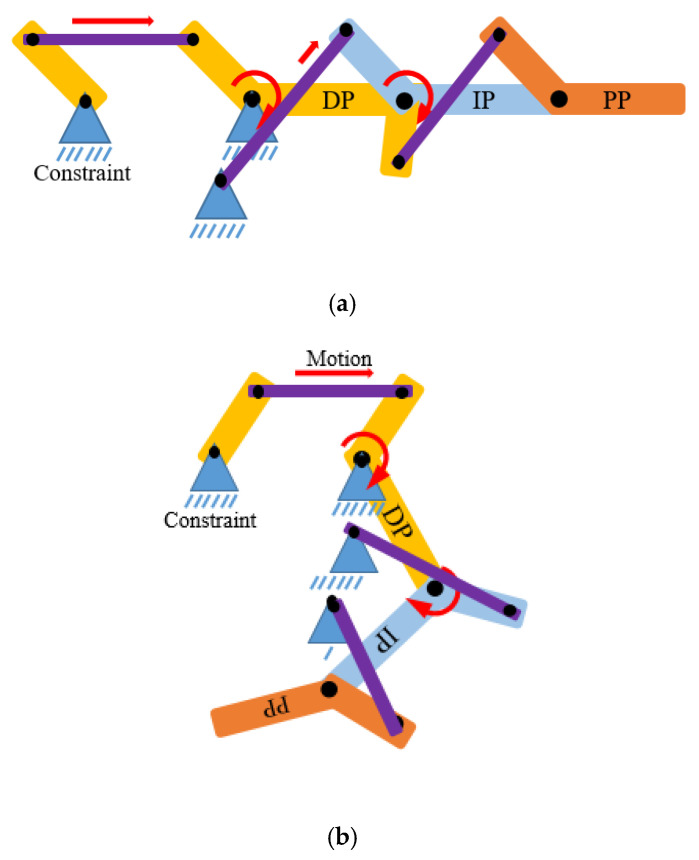
The finger robot structure: stick diagram (**a**) extension motion, (**b**) flexion motion.

**Figure 5:**
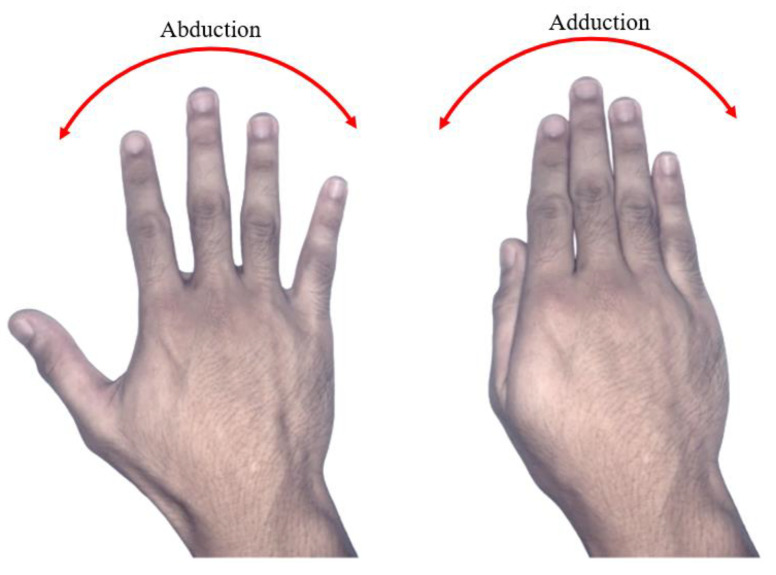
Abduction and adduction finger motion.

This section shows the specification of the structural design of the robot hand, including the limitation and improved features applied to the design, as listed below:

- Using the solid connector that connects the actuator and the robot structure to move the joint called planar four-bar linkage mechanism. Planar quadrilateral four-bar linkage are constructed from four links connected in a loop by four degrees of freedom. A link that grounds and connects the input and output links called the fixed link. The input and the output links are called the grounded links and it also can be classified into several classes such crank or rocker link. The crank link revolves around the grounded joint at about 360° while rocker link rotates the grounded joint at a limited range of angles. The other link, which connects the input and output links, and opposes the fixed link, is called the coupler. This link allows the motion generated from the input link to be transferred to the output link. In the robot design, both input and the output links are classified as rocker and are known as the double-rocker type. There are three double-rocker mechanisms needed in a complete finger, which are connected in series to connect four structure parts called the actuator—structure, DP, IP, and PP. These four structure parts are combined to become a complete finger structure [29]. The extension and flexion motion are represented in a four bar mechanism diagram and a stick diagram as shown in Figure 3 and Figure 4. In this research, each of the finger joints are dependent on each other, thus their phalanxes motion are correlated for each finger. This dependent finger joint concept is used to reduce the complexity and size of the system due to each finger only requiring an actuator to move against the system, whereas independent joints require multiple actuators for each finger. This concept is widely used in all of the robot hand designs as listed in Table 1. The four-bar linkage under-actuated concept was suggested to improve the drawbacks of the cable-driven under-actuated mechanism in terms of its motion range accuracy capability and durability [10,11].

- The bone length is a significant part that affects the finger structure size. The adult human hand is used as a reference to the structural design of the robot hand structure [5]. The length of the finger bones details, as shown in Table 2. The index, middle, ring, and baby finger are assumed to have a similar bone length.

- The motion range of the finger joints is shown in Table 3. The index, middle, ring, and baby fingers are assumed to have a similar motion range.

- The abduction and adduction motion of the actual finger, as shown in Figure 5, is excluded from the robot hand structure design.

### 2.2. Design

#### 2.2.1. Structural Design

**Figure 6:**
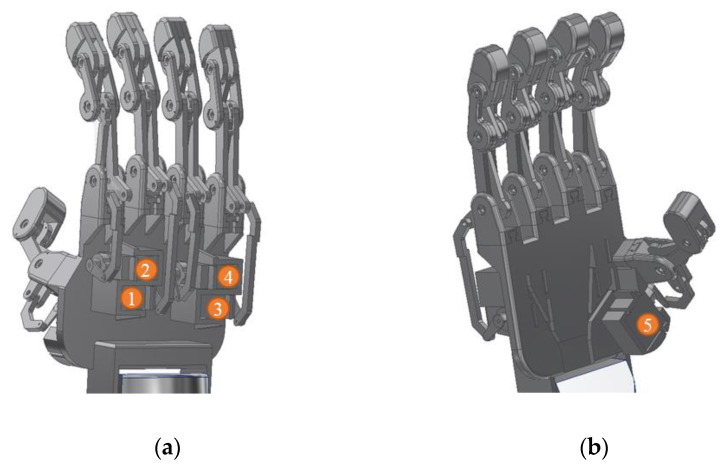
The structural design of the robot hand and the actuator position (1–5), (**a**) back view and (**b**) front view.

The structural design was constructed by applying the four-bar linkage mechanism and all the improved features listed in Table 1, such five fingers, complete finger joint, individual actuated finger, follows the average human hand size, applies the mechanical stopper, detachable finger structure and 3D printed structure. The design was drawn by using the Autodesk Inventor Professional 2018 software. The structural design of the robot hand structure was as shown in Figure 6. It consisted of five fingers, and an actuator was allocated for each finger to move the finger individually. The actuators for the index, middle, ring, and baby fingers were located at the back of the robot hand structure, as shown in Figure 6a. The actuator for the thumb was located at the front of the robot hand structure, as shown in Figure 6b.

**Figure 7:**
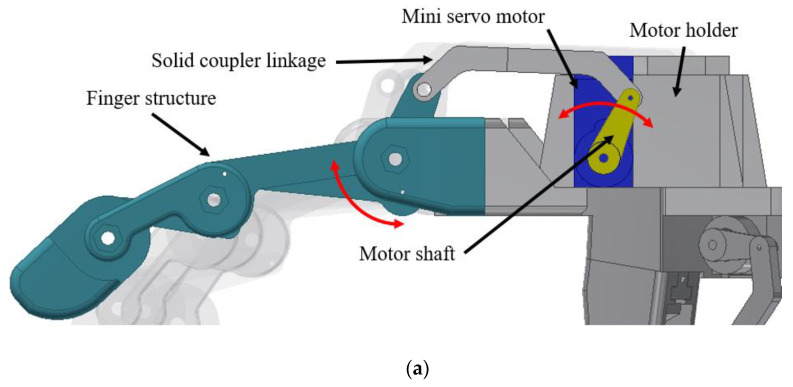

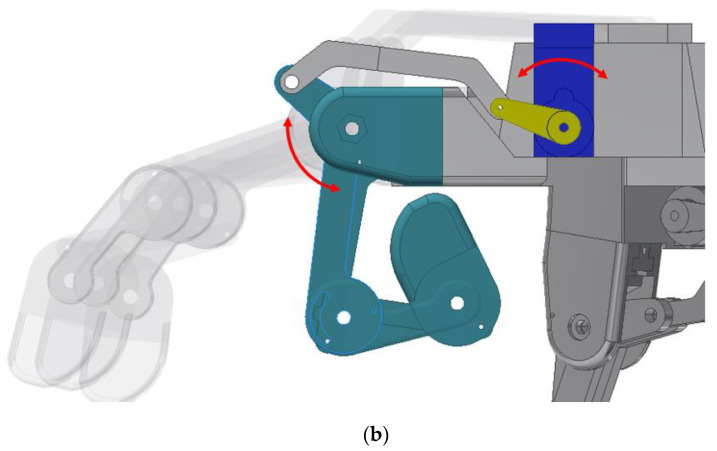
Finger actuation system: (**a**) extension and (**b**) flexion.

Each of the fingers had an actuator as shown in Figure 7. The actuator shaft was coupled with the finger using a solid coupler linkage which was to allow each finger move independently according to its motor shaft motion. The actuator used in this design was a servo motor due to its capability to control the motion angle. The extension and flexion motion of the finger structure are shown in Figure 7a,b, respectively. Solid coupler linkage was used to overcome the cable driven actuator in terms of the motion accuracy and durability issues.

**Figure 8:**
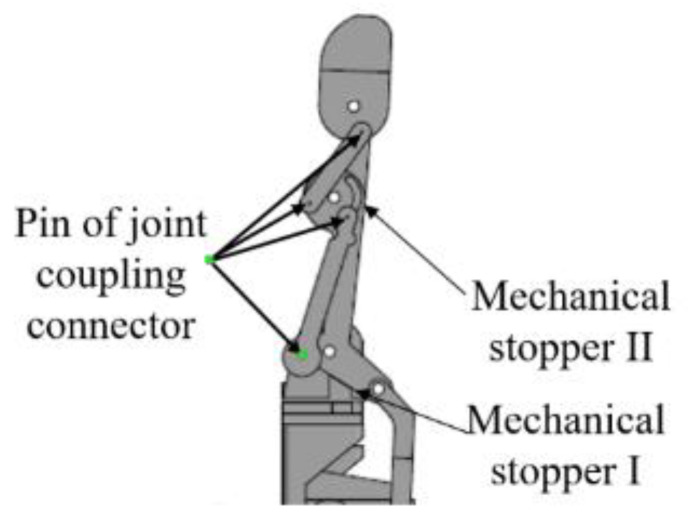
The overall design of the mechanical stopper of the finger structure.

In order to limit the motion range of the finger joint, the mechanical stopper feature was applied to the structural design. There were two mechanical stoppers, namely I and II, as shown in Figure 8. The mechanical stoppers were also used to protect the structure from being damaged when the excess load was exerted on it and excessive joint motion drawn by the actuator. In addition, the structural design for each finger had three joints per finger except the thumb that had two joints only. Therefore, the total number of joints in this robot hand was 14.

**Figure 9:**
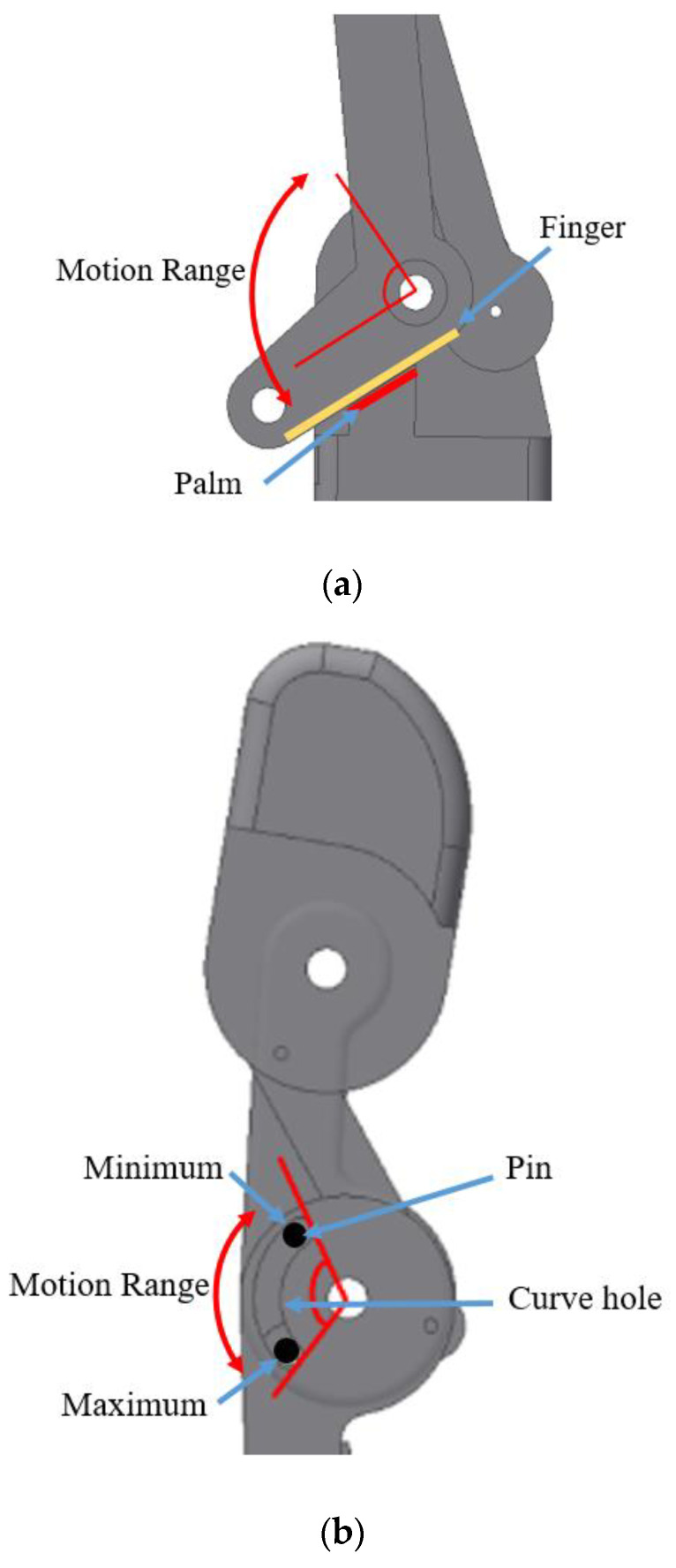
The mechanical stopper of the finger structure (**a**) mechanical stopper I; (**b**) mechanical stopper II.

The mechanical stopper I was used to limit the pull motion done by the actuator. The motion would be stopped when the finger structure hit the palm structure as shown in Figure 9a. The mechanical stopper II was used to limit the maximum and the minimum motion exerted on the fingertip by allowing the pin to move along the designated curve hole as shown in Figure 9b.

**Figure 10:**
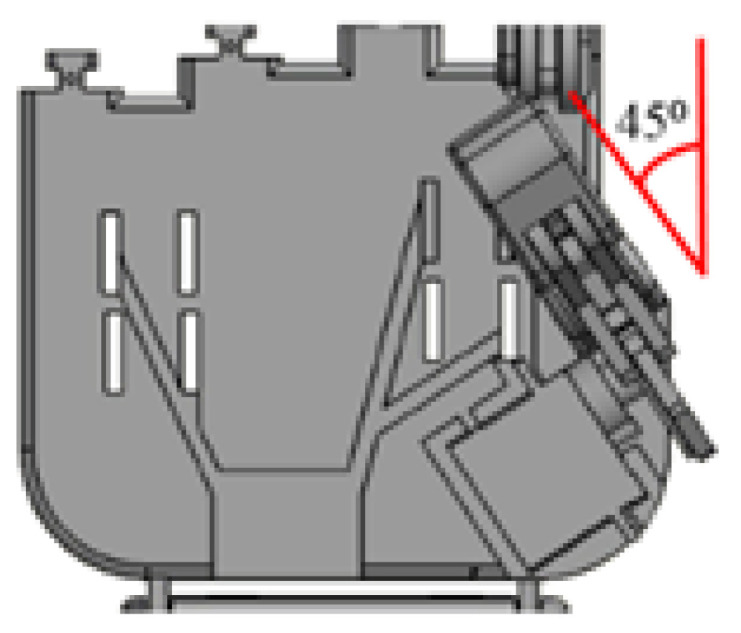
The thumb of the robot hand tilted 45° to the left.

**Figure 11:**
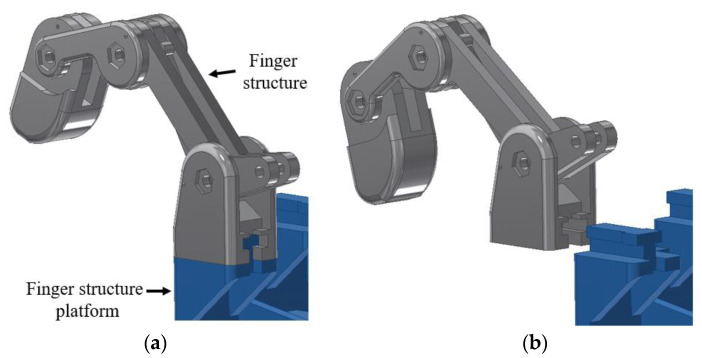
The detachable finger structure (**a**) attached to the palm and (**b**) detached from the palm structure platform (palm frame).

Figure 10 shows the thumb tilted 45° to the left, and perpendicular to the hand palm. This feature was applied to the structural design to improve the grip of the grasping posture. The detachable finger structure allowed the finger to be attached and detached from the palm structure to allow the user to replace the finger easily as shown in Figure 11a,b, respectively.

#### 2.2.2. Electrical and Electronic System

**Figure 12:**
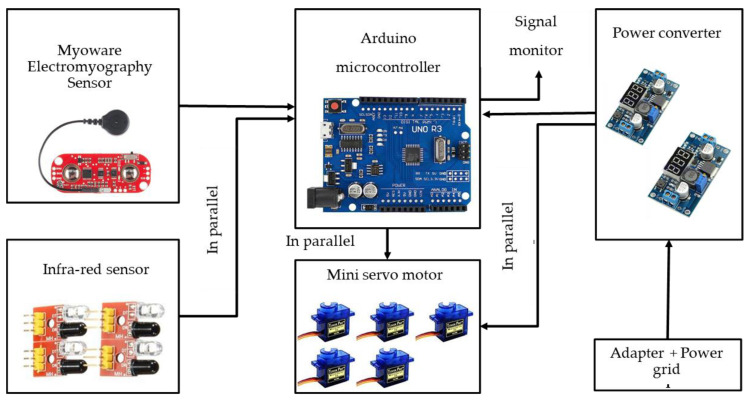
Electrical and electronic hardware configuration.

The electrical and electronic hardware configuration of the system and the specification of the components are shown as in Figure 12 and Table 4, respectively. The Myoware electromyography (EMG) sensor was used to detect the muscular activity of the user. The muscular activity (flexion and extension motion) was detected and used to control the hand robot system. Besides, four units of infra-red sensors were used as additional sensors to detect the presence of objects on the palm. In addition, there were five units of the mini servo motors to operate the five-finger structure independently. The mini servo motors were connected in parallel to the microcontroller. Thus, the output signal from the microcontroller for each motor could be generated simultaneously. The signals could be monitored on the personal computer (PC) monitor by connecting the USB port of the microcontroller to the PC. The power supply of the system was supplied from the power grid, and it was connected to the power converter to distribute the 5 V and 4 A of the power supply to the microcontroller and the mini servo motors.

### 2.3. Structural Design Evaluation

#### Static Analysis

Static analysis is the durability evaluation, which calculates the safety factor of the structure on the specific area by using the Autodesk Inventor Professional 2018. It is the ratio between the structure strength and the force with direction exerted on it. The colour region can be observed, and it is used to indicate whether the structure can withstand the static load or not. The green yellowish to red colour region is a dangerous condition (safety factor lower than 1), while the blue to green colour region represents that the structure can withstand the load (safety factor greater than 1).

Static load measurement is a crucial process that needs to be carried out in this kind of study. In the static load test, the load that can withstand the finger structure is investigated. Durability of the structure is one of the important aspects that must be take into account in order to develop a long lasting and safe device. Furthermore, a durable finger structure will be able to allow the application of high torque actuator in the robot system without getting any structure damage, and whether the actuator can be changed depends on the needs of the application, so the grasping force may be varied depending on the torque of the actuator used.

**Figure 13:**
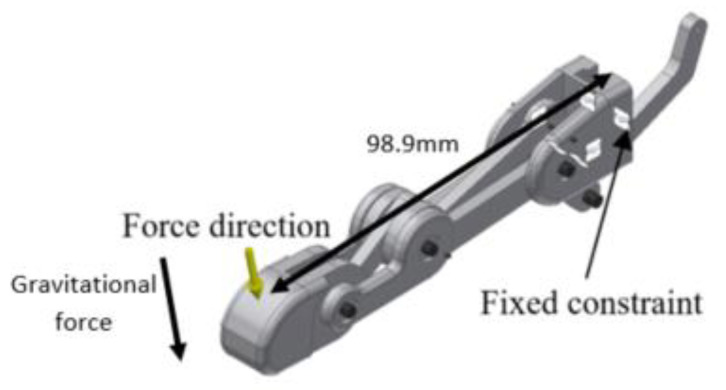
Experimental setup of the static analysis.

The material of the structure used in the experiment was the Acrylonitrile Butadiene Styrene (ABS) thermoplastic. The experiment was set up as shown in Figure 13. The finger structure was faced upward, and the load was applied incrementally by 100 g on it with a downward direction at the tip of the finger, which was about 98.8 mm from the structure constraint. The strength properties such the yield strength and tensile strength of the ABS material were $2.901\times 10^{3}\;psi$ and $4.293\times 10^{3}\;psi$, respectively.

### 2.4. Structure Fabrication

#### 3D Printing Technology Method

##### Specification of the 3D Printed Product

Table 5 lists the parameters that have been determined for the fabrication process of the 3D printed robot hand structure components.

### 2.5. 3D Printed Structure Evaluation

#### 2.5.1. Motion Capture Analysis

**Figure 14:**
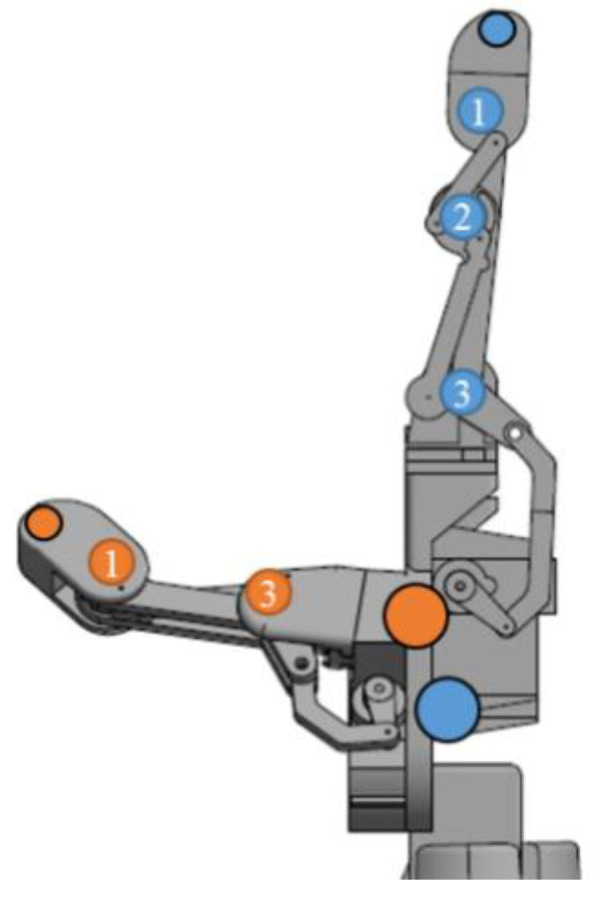
The placement of reflective markers on the robot hand structure: 1 = Distal Interphalangeal, 2 = Proximal Interphalangeal, and 3 = Metacarpophalangeal. Orange dot = thumb and blue dot = index finger.

**Figure 15:**
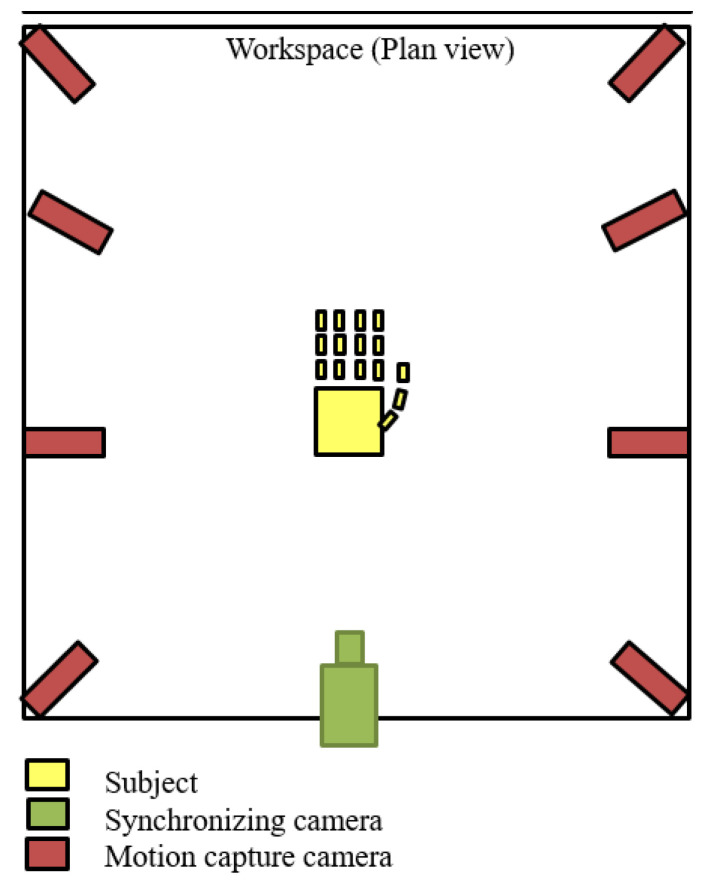
Experimental setup of the motion capture experiment.

The motion capture analysis is a method that can be used to measure and analyse the motion of actual human body parts accurately. This method is found to be able to give the most accurate result as compared to that of other existing methods [30]. However, this method has never been employed to measure and analyse robot hand motion. The experiment based on the motion capture analysis was conducted and the results were used to measure the similarity achieved by the constructed structure and the structural design. The maximum and minimum angles of each joint were measured, and their difference was calculated to obtain the motion range achieved by the joints. The structure was controlled to perform opened hand palm and the grasping posture for 2 s alternately, for three times for one samples. Five samples/sets were recorded with a sampling rate of 100 Hz by using 3D Venus software. The equipment used for this experiment was eight units of OptiTrack Flex 13 motion tracking cameras, a unit of Basler acA640-120gs synchronising camera and nine units of passive reflective markers. The reflective markers were placed as shown in Figure 14, and the experimental setup is shown in Figure 15. The structure was placed in the middle of the workspace and all the cameras were facing it.

#### 2.5.2. Load Test

**Figure 16:**
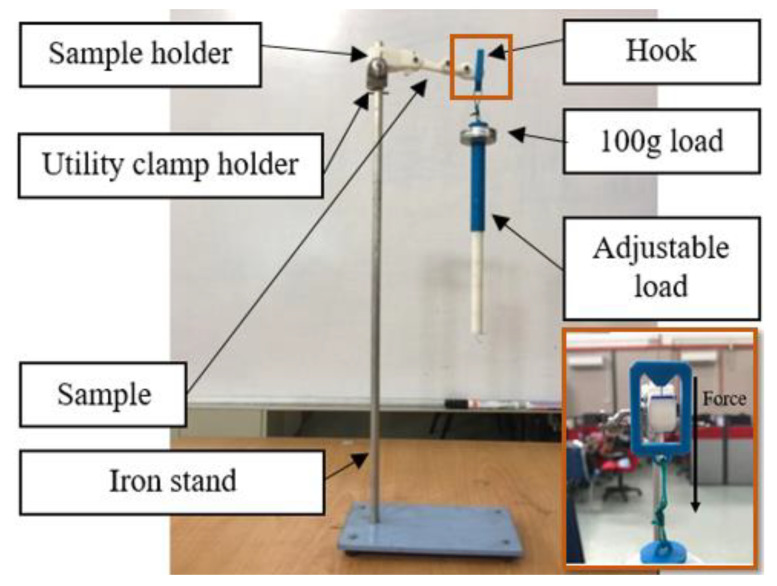
Experimental setup of the load test.

The load test is a method employed in this study to measure the durability of the actual structure, which is used to validate the simulation result obtained in Static analysis. However, the test that was done on the 3D printed robot hand built with Nylon material was used in the previous study [31], while the Acrylonitrile Butadiene Styrene (ABS) thermoplastic material was used in this experiment. An adjustable load hung on the tip of the finger structure, which is about 98.88 mm apart from the structure constraint. The structure faced upward, and the load was exerted downward, as shown in Figure 16. The load was increased incrementally by 100 g during the experiment until the structure started to break. Three samples with similar fabrication process were tested in the experiment to get an accurate result in the durability test.

## 3. Results and Discussion

### 3.1. Static Analysis

**Figure 17:**
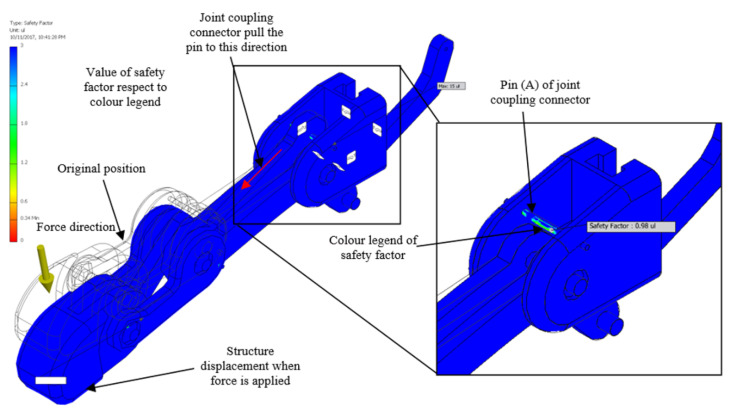
Simulation result of the static analysis towards Pin (A) of the joint coupling connector (JCC) of the robot structure made of Acrylonitrile Butadiene Styrene (ABS) material.

From the observation of the simulation result obtained, Pin (A) of the joint coupling connector (JCC) is found to be the weakest component of the robot structure with a static analysis value, as shown in Figure 17. Any components of the structure that reached the safety factor lower than 1 are shown to be the weakest component. A safety level below 1 is an indicator of a structure starting to break.

**Figure 18:**
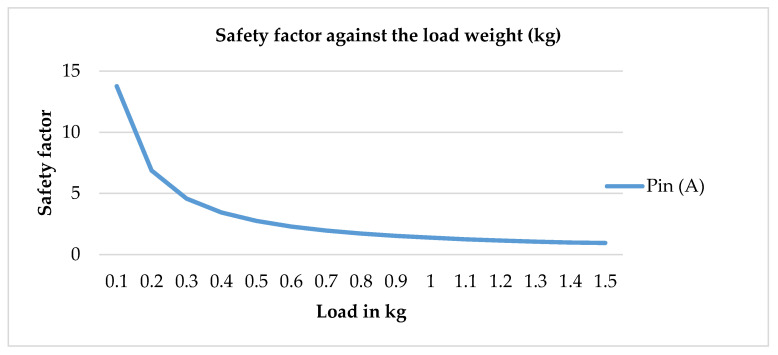
Safety factor value of the weakest component against the load weight graph.

The graph in Figure 18 illustrates the safety factor value of the Pin (A) of the JCC of robot hand structure of the ABS material against the load weight with an increment of 0.1 kg. In contrast, the safety factor of the Pin (A) of JCC is decreased exponentially along the graph. As a result of static analysis, the structure made of ABS started to break at 1.4 kg of the load.

### 3.2. 3D Printed Robot Hand Structure

**Figure 19:**
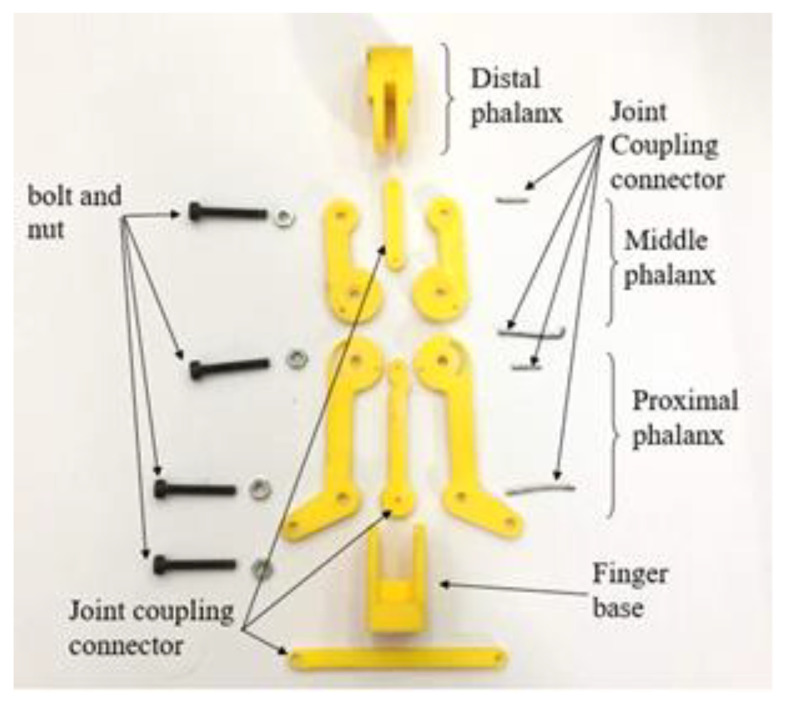
Components of a finger structure of the robot hand.

**Figure 20:**
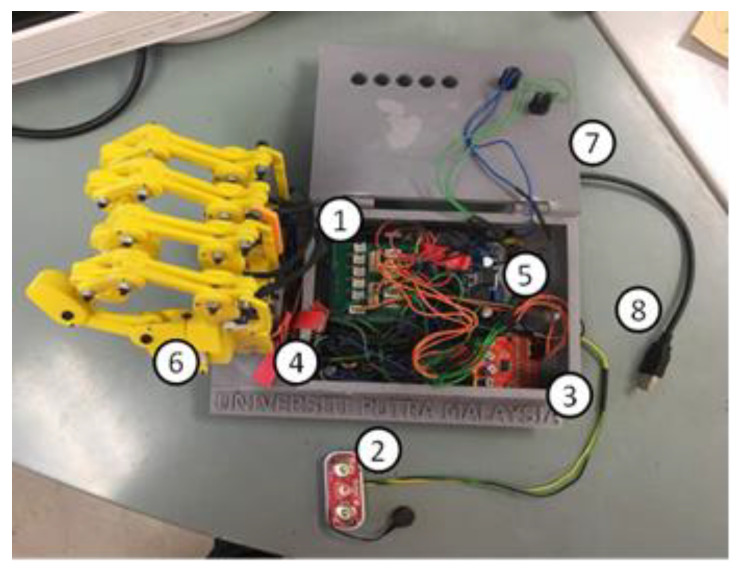
Complete prototype (**1**) Arduino Uno board, (**2**) MyoWare electromyography sensor, (**3**) Infrared sensor, (**4**&**5**) DC–DC converter, (**6**) robot hand structure, (**7**) buttons, and (**8**) power supply adaptor.

The components of the finger structure are made up of 3D printed parts (using ABS material) as body structures and some steel parts such as bolts and nuts as joint connectors, as shown in Figure 19. Figure 20 shows the complete prototype of the robot hand; an integration of the structure and electrical and electronics parts.

### 3.3. Load Test

**Figure 21:**
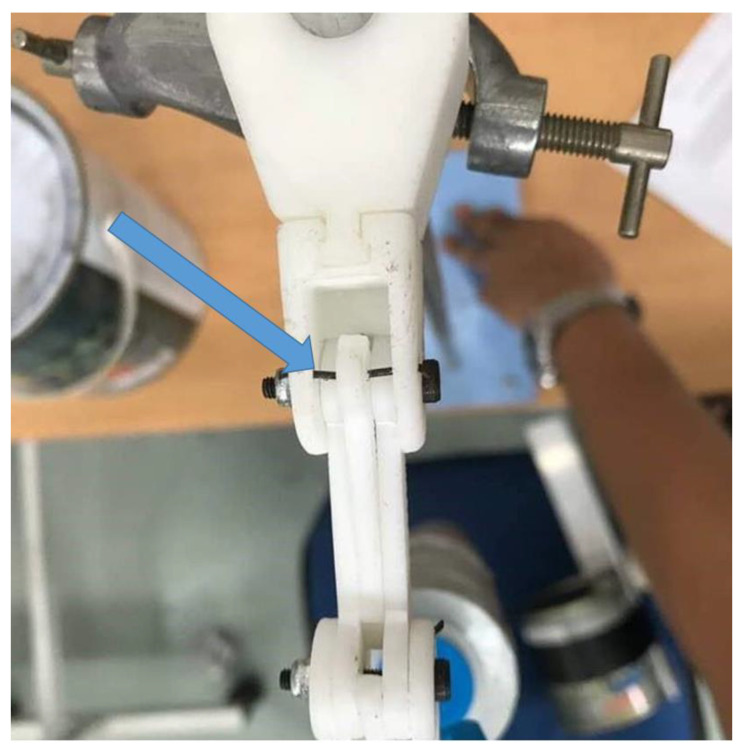
Bending effect of the pin (A) of JCC at maximum load for ABS structure.

Figure 21 depicts a noticeable change in Pin (A) of JCC when the pin on the structure made of ABS begins to bend at an average load weight equivalent to 1633 g. The experiment was extended, where additional loads were added up until the structure started to break. As a result, the ABS structure only withstood up to 2.7333 kg as shown in Table 6. The results obtained from the load test are close to the results obtained in the simulation (Static analysis), and the differences between the two are not significant; the load test results are approximately 200 g more than the simulated result.

### 3.4. Motion Capture Analysis

This analysis was performed to ensure that the movement capabilities of the 3D printed robot hand structure reached the optimum equilibrium with the expected range of motion as determined in the structural design. However, a limitation was identified during this experiment in which the reflective markers could not be affixed to all fingers except for the thumb and index finger due to insufficient space on the other finger structures. Thus, the reflective markers were only placed on the index finger and the thumb. As the index finger shares the same and identical structure as the middle, ring and baby fingers, these fingers were assumed to be similar to the index fingers. By referring to Table 7, the ROM similarity achieved is about 72.62–98.43%. The lowest ROM similarity occurred at the PIP joint of the index finger, whereas the highest ROM similarity occurred at the DIP joint of the finger. However, the similarity percentage of the ROM motion range obtained is still satisfactory.

### 3.5. Individual Controlled of the Finger

**Figure 22:**
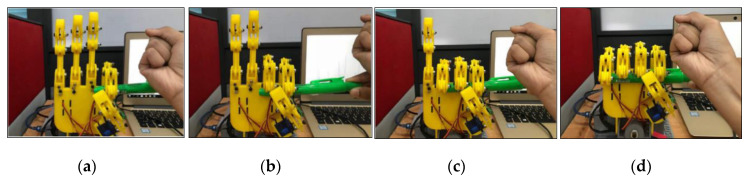
Motion of the robot hand for small cylinder object (**a**) Thumb and Index finger movement, (**b**) Thumb, Index and Middle finger movement, (**c**) Thumb, Index, Middle and Ring finger movement, and (**d**) All fingers movement.

**Figure 23:**
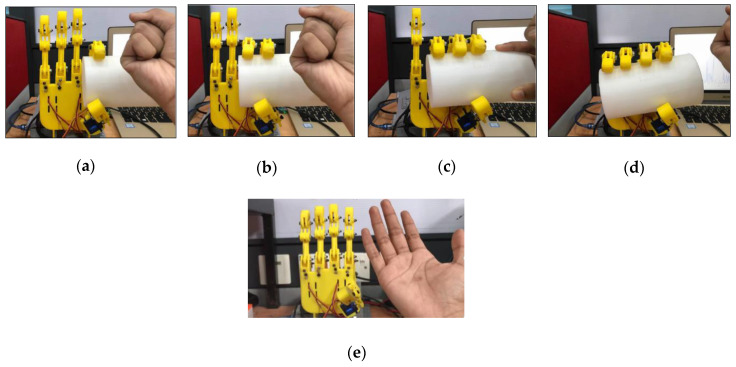
Motion of the robot hand for large cylinder (**a**) Thumb and Index finger movement, (**b**) Thumb, Index and Middle finger movement, (**c**) Thumb, Index, Middle and Ring finger movement, (**d**) All fingers movement and (**e**) Opened hand palm.

This experiment investigated the capability of the fingers where each finger was controlled individually. In this experiment the robot hand was tested to grasp a small and a large cylindrical object in order to demontrate that the finger can be controlled individually as shown in Figure 22 and Figure 23.

## 4. Conclusions

The 3D printed robot hand prototype was developed, with improved features of the structural design, such as a four-bar linkage mechanism, the same size as an average human hand, five-fingers with completed joints where each finger was moved by a motor individually, joint protection using a mechanical stopper, and a detachable finger structure from the palm frame features. The movement capability and durability were validated through motion capture analysis, static analysis, and load test. A similarity of 70–98% between the 3D design and the built robot structure was achieved, and it could withstand up to 1.6 kg load in both the simulation and the real test.

## 5. Future Works

**Figure 24:**
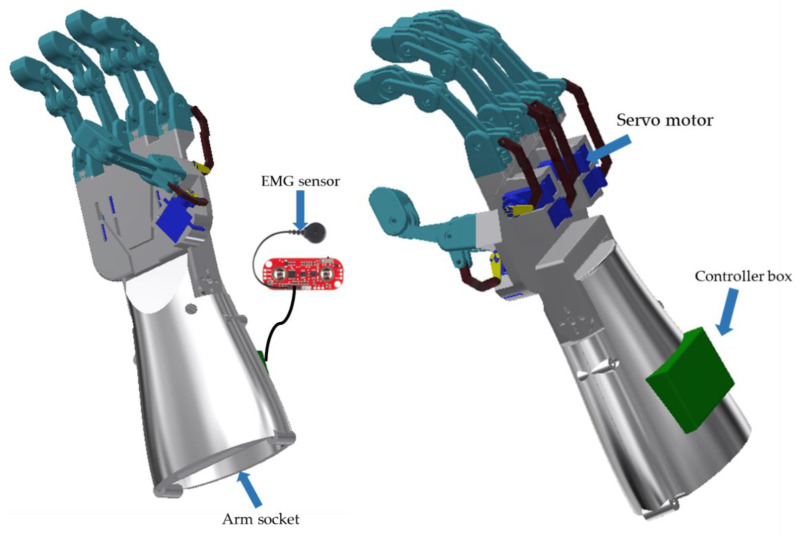
The robot hand design with arm structure for the prosthetic used.

The robot hand design with arm socket feature shown in Figure 24 is designated for the trans-radial amputation type. The arm socket structure is used to attach the robot structure to the patient arm. The arm socket size is designed based on the patient’s arm and can be produced easily with 3D printing technology. The robot hand can be controlled by attaching the EMG sensor on the skin near to the targeted muscles to control the hand motion. Besides, the controller box is used to store the electrical hardware including the battery (mobile power source). Due to these features, the mobile robot hand can be used for prosthetic use.

## Figures and Tables

**Table 1:** General characteristic of the existing robot hands.

Reference	Complete Finger (5)	Complete Joint (14)	Cable-Driven Actuator	Individual Actuated Finger	Average Human Hand Size	3D Printed Structure	Detachable Finger Structure	Mechanical Stopper
**Commercial device**								
**Vincent hand** [16]	✓	X	X	✓	✓	X	X	X
**iLimb hand** [17]	✓	X	X	✓	✓	X	X	X
**iLimb pulse** [17]	✓	X	X	✓	✓	X	X	X
**Bebionic hand** [18]	✓	X	X	✓	✓	X	X	X
**Bebionic V2** [18]	✓	X	X	✓	✓	X	X	X
**Michelanglelo hand** [19]	✓	X	X	X	✓	X	X	X
**Research prototype**								
**Prototype A** [4]	X	✓	X	X	X	X	X	X
**Prototype B** [5]	X	X	X	✓	X	✓	X	X
**Prototype C** [6]	X	✓	✓	X	X	X	X	X
**Prototype D** [8]	X	X	✓	X	✓	X	X	X
**Prototype E** [9]	✓	✓	✓	✓	X	X	X	X
**Prototype F** [10]	X	✓	✓	✓	X	✓	X	X
**Prototype G** [11]	✓	✓	✓	✓	X	X	X	✓
**Prototype H** [12]	✓	X	X	✓	X	X	X	X
**Gifu hand II** [13]	✓	✓	X	✓	X	X	X	X
**Robonaut hand** [20]	✓	✓	✓	✓	X	X	✓	✓
**Prototype I** [21]	✓	✓	X	✓	X	X	X	X
**Prototype J** [22]	✓	✓	X	✓	X	X	X	X
**Gifu hand III** [23]	✓	✓	X	✓	X	X	X	X
**Prototype K** [24]	✓	✓	✓	✓	✓	X	X	X
**Prototype L** [25]	✓	✓	✓	X	X	✓	X	X
**Tact Hand** [26]	✓	X	✓	✓	✓	✓	X	X

**Table 2:** The length of the finger bones.

Bone	Fingers	
	Thumb	Index, Middle, Ring & Baby
**DP**	2.325 cm	2.325 cm
**IP**	-	2.400 cm
**PP**	3.960 cm	3.960 cm

**Table 3:** The motion range of the finger bones.

Bone	Fingers	
	Thumb	Index, Middle, Ring & Baby
**DIP**	85°	90°
**PIP**	-	105°
**MCP**	90°	85°

**Table 4:** The electrical and electronic components of the system.

Component	Specification
Myoware Electromyography sensor	Operating Voltage: 2.9 V–5.7 V
	Operating Current: 9 Ma–14 mA
	Output mode: EMG envelope and raw signal
S-LFS-4-4 ways Infrared (IR) Sensor	S-LFS-4-4 ways Infra-red sensor
	Operating voltage: 3.3 V–5 V
	Operating current: >1 A
Arduino Microcontroller	Microcontroller: ATmega328
	Operating Voltage: 5 V
	Operating Current: 1 A
	Input Voltage (Recommended): 7 V–12 V
	Input Voltage: 5 V–20 V
	Digital I/O pins: 14 (6 PWM, Output)
	Analog I/O pins: 6
	DC Current per I/O pin: 40 mA
	DC Current for 3.3 V: 50 mA
	Clock Speed: 16 MHz
	Flash Memory: 32 KB
	SRAM: 2 KB
	EEPROM: 1 KB
Mini Servo Motor	Operating Voltage: 4 V–7.2 V
	Operating Current: 220 ± 50 mA
	Speed Operation: 0.1 s/60° at 4.8 V
	Torque: 1.8 Kg*cm at 4.8 V
	ROM: 180°
Power Converter	Input DC: 3.0 V–35 V
	Output voltage: 1.5 V–35 V (Input must greater 1.5 V than output)
	Output current: 2 A max
	Conversion efficiency: 92%
	Minimum drop voltage: 1.5 V
Power grid and adapter	ACDC converter
	Operating Voltage: 240 V 50 Hz
	Output Voltage: 12VDC
	Output Current: 2 A

**Table 5:** The specification of the parameters for the 3D printed product.

Specification	Quantity
**Type of printer**	Fused deposition modelling
**Infill percentage**	30%
**Shell thickness**	Ten layers
**Infill type**	Rectilinear
**Infill line thickness (Nozzle hole diameter)**	0.2 mm
**Resolution**	X = 0.2 mm Y = 0.2 mm Z = 0.1 mm
**Filament ink**	1.75 mm UP Fila Acrylonitrile Butadiene Styrene (ABS)

**Table 6:** The load test results obtained from the three samples.

Material	ABS		
Sample No.	1	2	3
**Load when Pin (A) (kg) started to bend**	1.600	1.700	1.600
**Average load (kg)**	1.633		
**Load when the structure started to break**	2.700	2.800	2.700
**Average load**	2.7333		

**Table 7:** The range of motion (ROM) similarity between the fabricated finger joint and the structural design.

Joints	Thumb				
	Minimum Angle (Measured)	Maximum Angle (Measured)	Calculated ROM	Expected ROM	Similarity
**DIP**	76.1969°	159.8606°	83.6637°	85°	98.43%
**PIP**	NA	NA	NA	NA	NA
**MCP**	95.2821°	172.8265°	77.5444°	90°	86.16%
	**Index**				
	Minimum angle (Measured)	Maximum angle (Measured)	Calculated ROM	Expected ROM	Similarity
**DIP**	90.6170°	165.0229°	74.4059°	90°	82.67%
**PIP**	101.5059°	177.7636°	76.2577°	110°	72.62%
**MCP**	99.4107°	173.351°	73.9410°	85°	86.70%
